# Pleistocene Climate Oscillations and Geographic Barriers Shaped the Phylogeographic Structure of *Anaplecta omei* (Blattodea, Blattoidea, Anaplectidae) in Southern China: Evidence From Mitochondrial Genomes

**DOI:** 10.1002/ece3.73086

**Published:** 2026-02-12

**Authors:** Tunan Zhou, Jing Zhu, Chenhui Cao, Yanli Che, Zongqing Wang

**Affiliations:** ^1^ College of Plant Protection Southwest University Chongqing China; ^2^ Key Laboratory of Agricultural Biosafety and Green Production of Upper Yangtze River (Ministry of Education) Southwest University Chongqing China

**Keywords:** biogeography, cockroach, demographic history, divergence time, population genetic structure

## Abstract

*Anaplecta omei* is the only species of *Anaplecta* widely distributed in southern China yet the processes shaping its genetic structure and range remain unclear. We used 97 complete mitochondrial genomes from 24 distinct geographic populations to characterize the genetic diversity, population structure, and evolutionary history of *A. omei*. Phylogenetic reconstruction, genetic diversity assessment, and population structure analysis revealed high haplotype diversity but low nucleotide diversity and a clear phylogeographic structure comprising four well‐differentiated groups. Selection tests indicated no group‐specific shifts in selective pressure, suggesting that physical barriers rather than adaptive divergence underlie this structure. Divergence‐time estimates showed that interspecific splits among *Anaplecta* date to the Miocene, whereas major intraspecific divergences within *A. omei* occurred during the Pleistocene, and demographic analyses (neutrality tests, mismatch distributions, and Bayesian skyline plots) indicated that the divergence within *A. omei* was followed by late‐Pleistocene population expansions in regional refugia. Together, these results indicate that tectonic uplift, Pleistocene climate oscillations, and complex topography have jointly shaped the phylogeographic diversification and distribution of *A. omei*, shedding light on diversification processes in Blattodea inhabiting montane landscapes.

## Graphical abstract

We used 97 complete mitochondrial genomes from 24 distinct geographic populations to characterize the genetic diversity, population structure, and evolutionary history of *Anaplecta omei*. Our results indicate that tectonic uplift, Pleistocene climate oscillations, and complex topography have jointly shaped the phylogeographic diversification and distribution of *A. omei*, shedding light on diversification processes in Blattodea inhabiting montane landscapes.

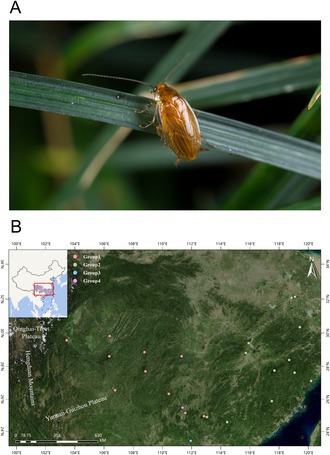

## 1 Introduction

**FIGURE 1:**
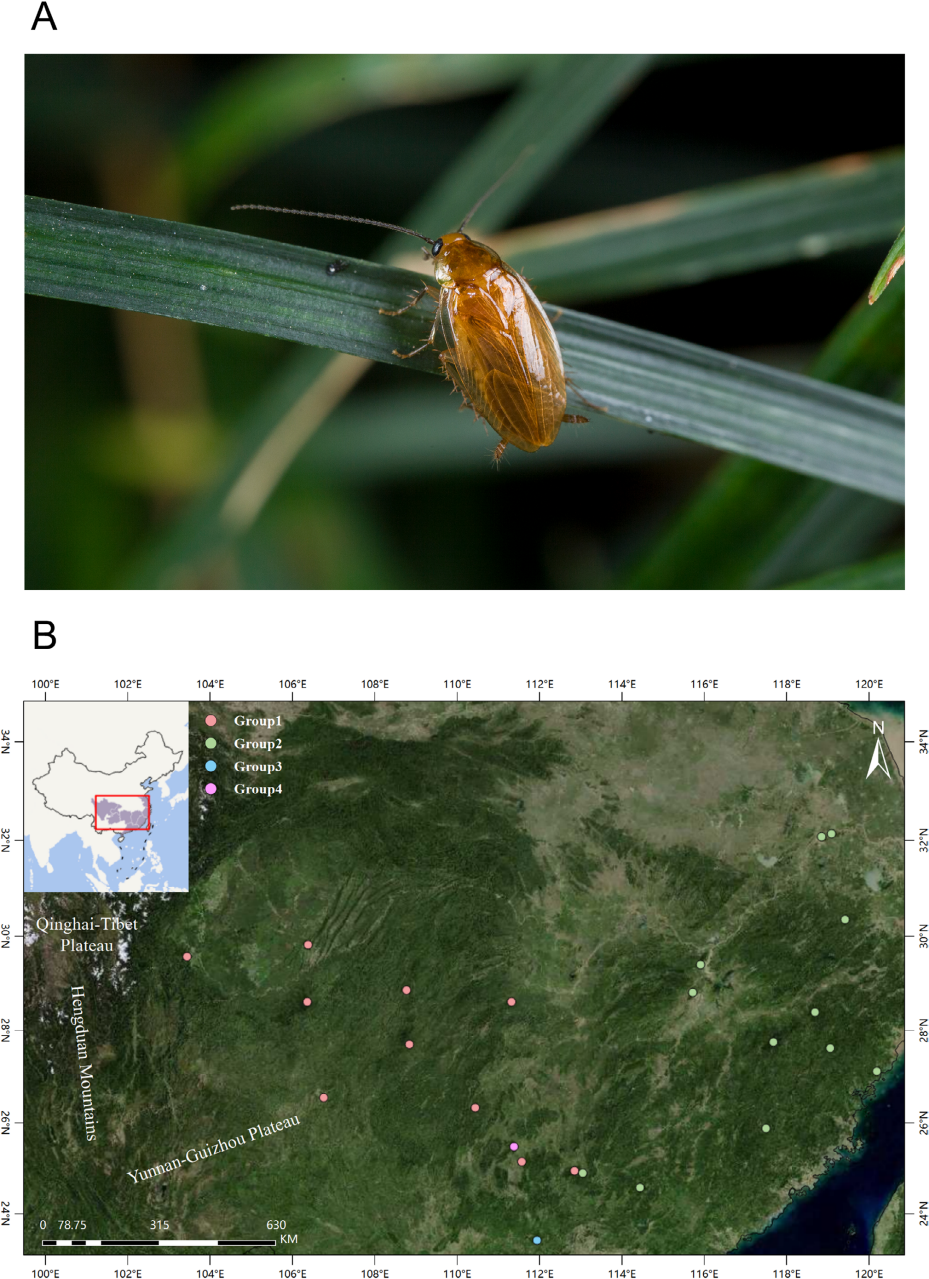
Field photograph and sampling map of *Anaplecta omei*. (A) *Anaplecta omei* perched on a blade of grass at night. Photograph by Lu Qiu. (B) Map showing the 24 sampling locations of *Anaplecta omei* in southern China.

*Anaplecta* Burmeister, 1838 (Blattodea, Blattoidea, Anaplectidae) is a small‐sized cockroach genus comprising 115 described species globally, including 25 recorded species in China (Djernæs et al. 2015; Wang et al. 2017; Beccaloni 2023). Based on more than a decade of field collection and behavioral observations by our research team, species of *Anaplecta* are primarily nocturnal insects that typically inhabit leaf surfaces or dense grassy areas and usually remain concealed within the leaf litter, shallow soil layers, or piles of stones near water bodies during the daytime (Figure 1A, T. Zhou, pers. observ.). Field observations and sparse behavioral reports suggest that flight in *Anaplecta* individuals is both infrequent and of short distance (Roth 1990; T. Zhou, pers. observ.). In China, most *Anaplecta* species have relatively narrow distribution ranges (Deng et al. 2020, 2022; Zhu et al. 2022), suggesting that dispersal and range may be constrained in this genus. As omnivorous insects, *Anaplecta* species act as important decomposers in nature and contribute substantially to maintaining ecological balance. Their potentially limited dispersal capacity and dependence on specific environments suggest that historical geological and climatic processes may have left pronounced signatures on their genetic structure and distribution.

Recent investigations have indicated that *Anaplecta omei* Bey‐Bienko, 1958 is the only member of the genus *Anaplecta* that is widely distributed in southern China (Deng et al. 2020; Deng et al. 2022; Zhu et al. 2022; Figure 1B), in sharp contrast to other Chinese *Anaplecta* species, which exhibit much narrower distribution ranges. Zhu et al. (2022) identified three species from the Yunnan–Guizhou Plateau, which are morphologically similar to *A. omei* but genetically and anatomically (male and female genitalia) distinct. After examining the morphological characteristics of *A. omei*, including both male and female genitalia, and integrating these observations with DNA barcodes from different geographic populations, *A. omei* was treated as a single species (Zhu et al. 2022; T. Zhou, pers. observ.) despite the presence of subtle differences. In a low‐dispersal, leaf‐litter‐dwelling insect such as *A. omei*, the occurrence of morphologically conserved yet genetically differentiated groups across a topographically complex landscape is more likely consistent with long‐term isolation driven primarily by geographic barriers and historical climatic fluctuations, rather than by strong divergent selection on external morphology. In such taxa, vicariance associated with mountain uplift and repeated range contractions and expansions during Pleistocene glacial cycles is expected to generate pronounced phylogeographic structure even in the absence of conspicuous morphological divergence (e.g., Ye et al. 2016; Du et al. 2023). The diversification of crown‐*Anaplecta* species began in the Cretaceous (Deng et al. 2023), and their early evolutionary history predates the uplift of the Qinghai–Tibet Plateau (An et al. 2001), providing the background conditions for the evolution of *Anaplecta* species currently distributed in southern China. Subsequent uplift of the eastern margin of the Qinghai–Tibet Plateau and the formation of the Yunnan–Guizhou Plateau would thus have acted on already diversified *Anaplecta* lineages, potentially fragmenting populations and promoting regional endemism. The divergence time between *A. omei* and other *Anaplecta* species dates back to the end of the Oligocene (Deng et al. 2023), whereas more recent geological events and Pleistocene climatic oscillations are expected to have structured intraspecific groups and population connectivity across the elevational gradients and heterogeneous habitats of southern China.

Following the Oligocene, the Earth experienced frequent tectonic activity and climatic fluctuations that affected numerous taxa (e.g., lizards, frogs, butterflies, birds, plants, and cicadas) in southern China (Harrison et al. 1992; Li and Fang 1999; Kou et al. 2006; Favre et al. 2015; Liu et al. 2018). Early Pleistocene climate changes expanded the distribution ranges of insects adapted to montane habitats in southern China (Ye et al. 2016: riffle bugs; Liu et al. 2023: mute cicadas), whereas repeated Pleistocene climate oscillations and geographic isolation promoted insect diversification and evolutionary processes, collectively shaping species diversity and distribution (Du et al. 2023: assassin bugs; Lin et al. 2024: dobsonflies and fishflies). The Yunnan–Guizhou Plateau, located southeast of the Qinghai–Tibet Plateau and with an average elevation above 1000 m, is recognized as a hotspot for biodiversity formation and preservation due to its complex topography and environmental heterogeneity (Chen et al. 2011; Zhu et al. 2017). These features make southern China, and particularly the Yunnan–Guizhou Plateau, an ideal region for investigating how geological uplift, climatic oscillations, and local environmental complexity interact to shape species' phylogeographic patterns. Within this region, *A. omei* occurs from lowland humid habitats to montane environments on the Yunnan–Guizhou Plateau (Figure 1B), which is on the southeastern margin of the Qinghai–Tibet Plateau. Combined with deep river valleys and dissected terrain, the Yunnan–Guizhou Plateau likely created multiple opportunities for population isolation, range shifts, and secondary contact in ground‐dwelling insects such as *A. omei*.

Based on the above, we propose the following hypothesis: the uplift of the Qinghai–Tibet Plateau and Pleistocene climatic fluctuations acted synergistically and, through geographic isolation and natural selection, jointly promoted the formation of the current distribution pattern of *Anaplecta* species, particularly *A. omei*. The widely distributed *A. omei*, which exhibits morphological continuity with other *Anaplecta* species (Zhu et al. 2022), serves as an excellent model for studying speciation and phylogenetic divergence, and its geographic pattern holds substantial research significance. However, previous studies were hampered by limited sample sizes of *A. omei*, undermining their representativeness. Consequently, there remain gaps in research concerning the geographic population structure of *A. omei*, and it is still unclear whether distinct geographic populations exist or what their divergence times are. The potential influence of tectonic and climatic changes on population dynamics has also been unexplored. To address these gaps and test our hypothesis, we conducted extensive sampling of *A. omei* and congeneric *Anaplecta* species across southern China and performed comprehensive phylogeographic analyses.

In recent years, mitochondrial genomes have emerged as powerful tools for phylogeographic analysis, effectively revealing species' genetic structures, population dynamics, and demographic history (Frandsen et al. 2020; Zelada‐Mázmela et al. 2022; Palumbi et al. 2023). Although mitochondrial genomes have limitations in resolving biogeographic questions—such as their rapid evolutionary rate and the fact that they effectively represent a single locus (Godinho et al. 2008; Zink and Barrowclough 2008), they have nonetheless elucidated the mechanisms underlying population divergence and expansion across diverse taxa (Du et al. 2019: periodical cicadas; Kim et al. 2022: wild silkworms; De León et al. 2023: electric fishes; du Plessis et al. 2023: eurasian otters; and Oosting et al. 2023: australasian snappers). In this study, we sequenced 97 mitochondrial genomes from 24 distinct geographic locations of *A. omei* across southern China and used these molecular markers to characterize genetic diversity, resolve group structure, and estimate divergence times among populations. We further examine how geological events, Pleistocene climatic oscillations, and regional environmental complexity have shaped the present‐day distribution of *A. omei*. This phylogeographic investigation provides novel insights into the evolutionary processes and biogeographic determinants governing diversification within Blattodea.

## 2 Materials and Methods

### 2.1 Sample Collection and Data Set

We sequenced 97 *A. omei* specimens collected from 24 localities and 11 congeneric species from three localities in southern China. The geographic information is provided in Figure 1B and Tables 1 and 2. All voucher specimens are preserved in 100% ethanol at −20°C and deposited at the College of Plant Protection, Southwest University (SWU), Chongqing, China. Either the hind‐leg coxa or thoracic muscle tissue was used for DNA extraction using the TIANamp Genomic DNA Kit (DP304; TIANGEN, Beijing, China). Genomic DNA was isolated and subjected to whole‐genome shotgun sequencing at Nanjing Personal Biotechnology Co. Ltd. (China). Sequencing libraries were constructed using a TruSeq DNA sample preparation kit with an average insert size of 400 bp. Each library was sequenced on an Illumina NovaSeq platform with PE150, yielding ~3 Gb of raw data. Sequencing statistics of the mitochondrial genomes of *Anaplecta* are provided in Table S1. Low‐quality reads and short reads were removed with Trim Galore (http://www.bioinformatics.babraham.ac.uk/projects/trim_galore/). We employed the clean data and selected the published mitochondrial genome of *A. omei* (GenBank: ON645474) from NCBI as the homologous reference, based on its close phylogenetic relationship to our study species. The assembly of the mitochondrial genome was performed in Geneious Prime v2021 (Biomatters Ltd., Auckland, New Zealand; https://www.geneious.com) using the map‐to‐reference strategy. Circularization of the mitochondrial genome was validated by identifying a ~100 bp overlapping sequence at the start and end regions of the linear sequence. The assembled mitochondrial genomes were annotated in Geneious Prime v2021 using the map‐to‐reference function, with a previously published and annotated mitochondrial genome of *A. omei* from NCBI (GenBank: ON645474) serving as the reference. Transfer RNA genes were annotated with MITOS2 (http://mitos.bioinf.uni‐leipzig.de/index.py on the Galaxy platform) under the invertebrate genetic code (Bernt et al. 2013). Protein‐coding genes (PCGs) and ribosomal RNA genes were identified by alignment with homologous mitochondrial genes available in NCBI. The start codons were identified as either the canonical ATG or other initiation codons widely recognized in Blattodea insects. For the stop codons, we annotated both complete (TAA and TAG) and incomplete codons (e.g., TA‐ or T‐‐). For rRNA genes, the 16S rRNA was located between tRNA‐Leu^CUN^ and tRNA‐Val. The 3′ end of 12S rRNA was directly adjacent to the last base of tRNA‐Val. Alignments were generated using MAFFT v7 (https://mafft.cbrc.jp/alignment/server/) (Katoh et al. 2019). To account for the characteristics of different sequence types, distinct alignment strategies were employed: the G‐INS‐i algorithm, suitable for global alignment, was used for PCGs, while the Q‐INS‐i algorithm, which incorporates both primary sequence and secondary structure information, was used for non‐coding fragments (22 tRNAs, 12S rRNA, and 16S rRNA). Afterward, manual adjustments were made in MEGA v.7.0 (Kumar et al. 2016). Alignments of PCGs were corrected by translation into amino acids with their start and stop codon positions ultimately determined through this process. The remaining sequences were visually inspected, and obvious misalignments in intergenic regions were removed. Two data matrices were then prepared: (i) PCGs (13 protein‐coding genes) for selection‐pressure analyses and (ii) MG (13 PCGs +22 tRNAs +2 rRNAs) for all other analyses.

**TABLE 1:** Sampling information, genetic parameters, and diversity of each group of *A*. *omei* based on MG dataset.

Group	Population	Sampling location	Sample size	Voucher number	Accession number	Haplotype number *Nh*	Haplotype diversity *Hd*	Polymorphic sites *S*	Nucleotide diversity *π*
Group 1	CQBB	Jinyun Mountain, Beibei, Chongqing	5	SWU‐B‐AN0029‐33	PX672859‐63	5	1.000	41	0.00114
	CQSMS	Simian Mountain, Chongqing	3	SWU‐B‐AN0034‐36	PX672864‐66	3	1.000	3	0.00014
	CQTHY	Taohuayuan, Youyang, Chongqing	5	SWU‐B‐AN0037‐41	PX672867‐71	2	0.600	1	0.00004
	GZFJS	Fanjing Mountain, Guizhou	5	SWU‐B‐AN0067‐71	PX672900‐04	4	0.900	13	0.00041
	GZNM	Nanming, Guizhou	5	SWU‐B‐AN0072‐76	PX672912‐16	1	0	0	0
	HNCB	Chengbu, Hunan	5	SWU‐B‐AN0077‐81	PX672917‐21	5	1.000	5	0.00014
	HNJYS	Jiuyi Mountain, Hunan	2	SWU‐B‐AN0084‐85	PX672924‐25	2	1.000	19	0.00130
	HNMS	Mang Mountain, Hunan	4	SWU‐B‐AN0086‐89	PX672926‐29	3	0.833	64	0.00220
	HNWYJ	Wuyun Boundary, Hunan	5	SWU‐B‐AN0090‐94	PX672930‐34	5	1.000	25	0.00080
	SCEM	Emei Mountain, Sichuan	5	SWU‐B‐AN0117‐121	PX672957‐61	5	1.000	14	0.00052
Group 2	FJTBY	Tianbao Rock, Fujian	5	SWU‐B‐AN0042‐46	PX672872‐76	5	1.000	30	0.00100
	FJTLS	Taimu Mountain, Fujian	5	SWU‐B‐AN0047‐51	PX672877‐81	5	1.000	33	0.00100
	FJWYS	Wuyi Mountain, Fujian	6	SWU‐B‐AN0052‐57	PX672882‐87	3	0.733	6	0.00018
	GDSG	Shaoguan, Guangdong	4	SWU‐B‐AN0058‐61	PX672888‐91	1	0	0	0
	JSBHS	Baohua Temple, Jiangsu	5	SWU‐B‐AN0095‐99	PX672935‐39	4	0.900	14	0.00041
	JSNJ	Nanjing, Jiangsu	5	SWU‐B‐AN0100‐104	PX672940‐44	4	0.900	7	0.00027
	JXLN	Longnan, Jiangxi	2	SWU‐B‐AN0105‐106	PX672945‐46	2	1.000	25	0.00127
	JXNC	Nanchang, Jiangxi	5	SWU‐B‐AN0112‐116	PX672952‐56	5	1.000	23	0.00078
	JXLS	Lushan Mountain, Jiangxi	5	SWU‐B‐AN0107‐111	PX672947‐51	4	0.9000	12	0.00036
	ZJJS	Jiangshan, Zhejiang	1	SWU‐B‐AN0122	PX672963	1	—	—	—
	ZJQY	Qingyuan, Zhejiang	2	SWU‐B‐AN0123‐124	PX672964‐65	2	1.000	6	0.00041
	ZJTMS	Tianmu Mountain, Zhejiang	1	SWU‐B‐AN0125	PX672966	1	—	—	—
Group 3	GDQLS	Qilin Mountain, Guangdong	5	SWU‐B‐AN0062‐66	PX672892‐96	5	1.000	14	0.00044
Group 4	HNDPL	Dupangling, Hunan	2	SWU‐B‐AN0082‐83	PX672922‐23	2	1.000	19	0.00130
	ALL		97			79	0.994	529	0.00371

**TABLE 2:** Sampling information of *A. longihamata*, *A. condensa*, and *A. paraomei*.

Species	Population	Sampling location	Sample size	Voucher number	Accession number
*A. paraomei*	GZDS	Du Mountain, Guizhou	3	SWU‐B‐AN0126‐128	PX672897‐99
*A. condensa*	GZJO	JiaOu, Guizhou	2	SWU‐B‐AN0129‐130	PX672905‐06
	GZLB	LiBo, Guizhou	5	SWU‐B‐AN0131‐135	PX672907‐11
*A. longihamata*	YNSP4	Wuliang Mountain, Yunnan	1	SWU‐B‐AN0136	PX672962
	ALL		11		

### 2.2 Phylogenetic Analyses

We analyzed 110 mitochondrial genomes, including sequences from GenBank (ON645468, ON645477). The ingroup comprised 97 *A. omei* individuals from 24 localities in southern China, whereas the outgroup contained 13 sequences of five congeneric species: *A. longihamata* Zhu and Che 2022, *A. condensa* Zhu and Che 2022, *A. paraomei* Zhu and Che 2022, *A. strigata* Deng and Che 2020, and *A. arcuata* Deng and Che 2020.

For phylogenetic reconstruction, codon‐specific saturation was evaluated in DAMBE v7.2.136 (Xia et al. 2003; Xia and Lemey 2009). The third codon positions were highly saturated (*Iss* = 0.075) relative to the first and second positions (*Iss* = 0.019) and were therefore excluded. Optimal substitution models for each partition were selected in PartitionFinder v2.1.1 (Lanfear et al. 2017) under the corrected Akaike information criterion (AICc): TRN + I + G for ND4, ND5, ND1 and ND4L; TVM + G for ATP8, ND6 and ND2; GTR + I for COI; GTR + I + G for COII, COIII and CYTB; GTR + G for ND3, ATP6, 12S and 16S; and HKY + I + G for the tRNA set. Maximum‐likelihood (ML) was used in IQ‐TREE v2.1.3 (Nguyen et al. 2015). Node support values were estimated using 1000 normal bootstrap replicates (BS). Bayesian inference (BI) was used in MrBayes v3.2.5 (Ronquist et al. 2012). Partitioning strategies and substitution models were based on the PartitionFinder results mentioned above. Two independent Markov chain Monte Carlo were run for 10,000,000 generations, sampling every 1000 generations, with the first 25% of samples discarded as burn‐in. Convergence was assessed in Tracer v1.7.1 (Rambaut et al. 2018) by confirming that the effective sample size (ESS) values ≥ 200. Nodes with Bayesian posterior probabilities (BPP) ≥ 0.95 were considered to be strongly supported (Ronquist et al. 2012). The ML and BI trees were visualized in FigTree v1.4.2 (http://tree.bio.ed.ac.uk/software/figtree/), and a circular phylogram was generated using the Interactive Tree of Life (iTOL) website (Letunic and Bork 2021).

### 2.3 Genetic Diversity and Genetic Structure Analyses

Population genetic parameters including the number of haplotypes (*H*), the number of polymorphic sites (*S*), haplotype diversity (*Hd*), nucleotide diversity (*π*), and genetic differentiation coefficients (*Gst* and *Nst*) were calculated using DnaSP v6.12.03 (Rozas et al. 2017). *Gst* is calculated primarily based on haplotype frequencies, whereas *Nst* incorporates both haplotype frequencies and the genetic relationships among haplotypes. These two values can be used to determine whether haplotypes exhibit a significant phylogeographic structure.

Bayesian population structure was analyzed using BAPS v6.0 (Corander et al. 2008), with the spatial clustering of individuals module. We tested *K* values ranging from 2 to 10, and the *K* value corresponding to the maximum log‐likelihood value was considered the optimal grouping. Analysis of molecular variance (AMOVA) was completed in Arlequin v3.5 (Excoffier and Lischer 2010). *Fst* was estimated in Arlequin v3.5 to quantify the differentiation between pairwise populations and pairwise genetic groups, with statistical significance tested by 1000 non‐parametric permutations at the 5% significance level. The *Fst* value reflects the level of heterozygosity among populations and is used to measure the degree of population differentiation. Gene flow, quantified as the effective number of migrants per generation (*N*_*m*_), was calculated according to *Fst*, *N*_*m*_ = (1 − *Fst*)/4*Fst* (Wright 1949). We used the Codeml program in PAML v4.9 (Yang 2007) to test for selection across the PCG dataset, with branches defined according to BAPS grouping results. This approach allowed us to assess whether group divergence was accompanied by shifts in selective pressures. Substitution models for the PCG dataset were selected following the same criteria used in phylogenetic analyses (see Table S2 for model details). We implemented both the one‐ratio model (M0), which assumes a uniform dN/dS ratio (*ω*) across all branches, and the two‐ratio branch model, which allows different *ω* values for designated foreground and background branches (Yang et al. 2000). Haplotype networks were constructed using the median joining network algorithm in PopART v1.7 (Leigh and Bryant 2015).

### 2.4 Divergence Time Estimation

Divergence times of *Anaplecta* were estimated in BEAST v1.10.2 (Suchard et al. 2018) using the MG dataset, with the same substitution models as in the phylogenetic analyses. We performed preliminary analyses using an uncorrelated log‐normal relaxed clock and evaluated the results in Tracer v1.7.1 (Rambaut et al. 2018). Based on the value of the ucld.stdev parameter, we selected either a strict clock model (if ucld.stdev was very close to 0) or a relaxed clock model (if ucld.stdev was greater than 1) (Drummond et al. 2006). The molecular clock was calibrated with two secondary calibration points from Deng et al. (2023): the split between *A. strigata* and *A. arcuata* , 89.9 Ma (95% credibility interval, 71.1–108.1 Ma), and between *A. arcuata* and remaining *Anaplecta* species, 86.2 Ma (95% credibility interval, 69.9–101.6 Ma). These node ages were implemented as soft‐boundary priors.

We specified a Birth‐Death Process (Gernhard 2008) and a strict molecular clock (Brown and Yang 2011) in the analysis. Two independent runs of 50 million Markov chain Monte Carlo (MCMC) generations were performed, sampling every 5000 steps. Convergence was confirmed when ESS values exceeded 200 in Tracer v1.7.1 (Rambaut et al. 2018). The maximum clade credibility tree was summarized within TreeAnnotator v1.10.2.

### 2.5 Demographic History Analysis

Population demographic dynamics were reconstructed using neutrality tests, mismatch distribution analysis, and Bayesian skyline plots. Neutrality tests and mismatch distributions were calculated in Arlequin v3.5 using the MG dataset. Tajima's *D* and Fu's *Fs* statistics tested for signatures of demographic equilibrium versus expansion, with significantly negative values indicating population growth (Tajima 1989; Fu 1997).

Unimodal mismatch distributions suggest population expansion, whereas multimodal patterns indicate demographic equilibrium (Rogers and Harpending 1992). The sum of squared deviations (SSD) and Harpending's raggedness index (*r*) assessed goodness‐of‐fit between observed and expected distributions. The expansion time (*t*) was calculated as *t = τ*/2 μk, where *k* = 3409 bp (sequence length) and *μ* = 0.0177 (the substitution rate of COI gene) (Rogers and Harpending 1992). Finally, Bayesian skyline plot analysis was implemented in BEAST v.2 (Bouckaert et al. 2014) using the MG dataset with a relaxed lognormal clock and a mean substitution rate of 3.342% per site per million years. The best‐fitting partitioning schemes are detailed in Table S3. Analysis ran for 50 million MCMC generations, and then demographic trajectories were visualized in Tracer v1.7.1.

## 3 Results

### 3.1 Genetic Diversity and Population Genetic Structure

We sequenced 97 complete mitochondrial genomes of *A. omei* and 11 of congeneric *Anaplecta* species (GenBank accession numbers in Table S1). All 108 mitochondrial genomes comprised 37 functional genes and one control region. Detailed information on genome lengths, GC contents, and average nucleotide composition is provided in Table S1. Across the 97 genomes of *A. omei*, we detected 529 polymorphic sites, including 364 parsimony‐informative sites and 165 singletons. These variants defined 79 haplotypes, yielding overall haplotype diversity (*Hd*) of 0.994 and nucleotide diversity (*π*) of 0.00371 (Table 3). Among geographic populations, HNMS exhibited the highest nucleotide diversity (*π* = 0.00220) and relatively high genetic diversity (*Hd* = 0.833) (Table 1).

**TABLE 3:** Genetic diversity of each group of *A*. *omei* populations based on MG dataset.

Group	Sample size	Haplotype number *Nh*	Haplotype diversity *Hd*	Nucleotide diversity *π*
Group 1	44	35	0.938	0.00133
Group 2	46	37	0.987	0.00111
Group 3	5	5	1.000	0.00044
Group 4	2	2	1.000	0.00130
ALL	97	79	0.994	0.00371

*Note:* Values of *Hd* > 0.5 and *π* > 0.005 suggest high haplotype and nucleotide diversity, respectively.

The genetic differentiation coefficients showed *Gst* (0.15487) < *Nst* (0.82952), with a statistically significant difference (*p* < 0.05). This result indicates that the haplotype distribution in *A. omei* exhibits a significant phylogeographic structure. BAPS clustering indicated that the log‐marginal likelihood increased with *K* and stabilized at its maximum at *K* = 4. The test results for additional *K* values can be found in Figure S1A. Accordingly, four groups were identified as the optimal population partition: Group 1, comprising populations mainly from Sichuan Province, Hunan Province, and Chongqing; Group 2, consisting largely of populations from southeastern China (Guangdong, Fujian, Jiangsu, Zhejiang, and Jiangxi Provinces); Group 3, containing only the Guangdong population (GDQLS) and Group 4, comprising only the Hunan population (HNDPL) (Table 1, Figure S1B). The genetic diversity of each group of *A*. *omei* populations based on the MG dataset is presented in Table 3. Branch‐model tests in PAML showed that the single‐ratio model (M0) estimated a genome‐wide nonsynonymous/synonymous substitution rate ratio (*ω*) of 0.12368 for all *A. omei* populations, consistent with strong purifying selection. Likelihood‐ratio tests comparing the two‐ratio model with M0 were not significant, indicating no detectable group‐specific differences in selective pressure (Table 4).

**TABLE 4:** Group‐specific estimates of *ω* and likelihood‐ratio statistics for *A. omei* under alternative branch models.

Foreground branch	*ω* (dN/dS) value		*p* (likelihood *p*)
	One‐ratio model	Two‐ratio model (B/F)	
Group 1	0.12368	0.12515/0.06947	0.56850
Group 2		0.12527/0.08609	0.63391
Group 3		0.12962/0.10427	0.51430
Group 4		0.12443/0.10829	0.85456

AMOVA analysis (Table S4) showed that most of the variation was distributed among groups (77.02%), with less variation among populations (12.68%) and within populations (10.30%). Moreover, the pairwise *Fst* values among groups ranged from 0.6895 to 0.9495, indicating very high inter‐group differentiation. The lowest inter‐group *Fst* value (0.68946) was observed between Group 1 and Group 2, which may be influenced by the limited sample sizes of Group 3 and Group 4; nonetheless, substantial genetic differentiation was evident among all groups. The pairwise *Fst* values among populations ranged from 0.0638 to 1.0000, with most values exceeding 0.25 (Table 5, Figure S2). The maximum gene flow (*N*_*m*_) among groups was 0.1126, and only 2% of population pairs showed *N*_*m*_ > 1 (Figure S2). Relatively frequent gene exchange was observed between population JXLN and populations ZJJS and ZJTMS (*N*_*m*_ up to 3.667), much higher than other population pairs. Overall, these results indicate significant genetic differentiation within *A. omei*.

**TABLE 5:** Genetic differentiation index (*Fst*) and gene flow (*N*_*m*_) among different geographical groups of *A. omei*.

Group	Group 1	Group 2	Group 3	Group 4
Group 1	0	0.1126	0.02645	0.08554
Group 2	0.6895	0	0.02176	0.06419
Group 3	0.9043	0.9199	0	0.01333
Group 4	0.7451	0.7957	0.9494	0

*Note:* Non‐shaded areas represent *Fst* values, shaded areas indicate *N*_*m*_ values. *Fst* values > 0.25 and *N*_*m*_ values > 1 indicate significant genetic differentiation and frequent gene flow, respectively.

### 3.2 Phylogenetic Constructions and Networks

**FIGURE 2:**
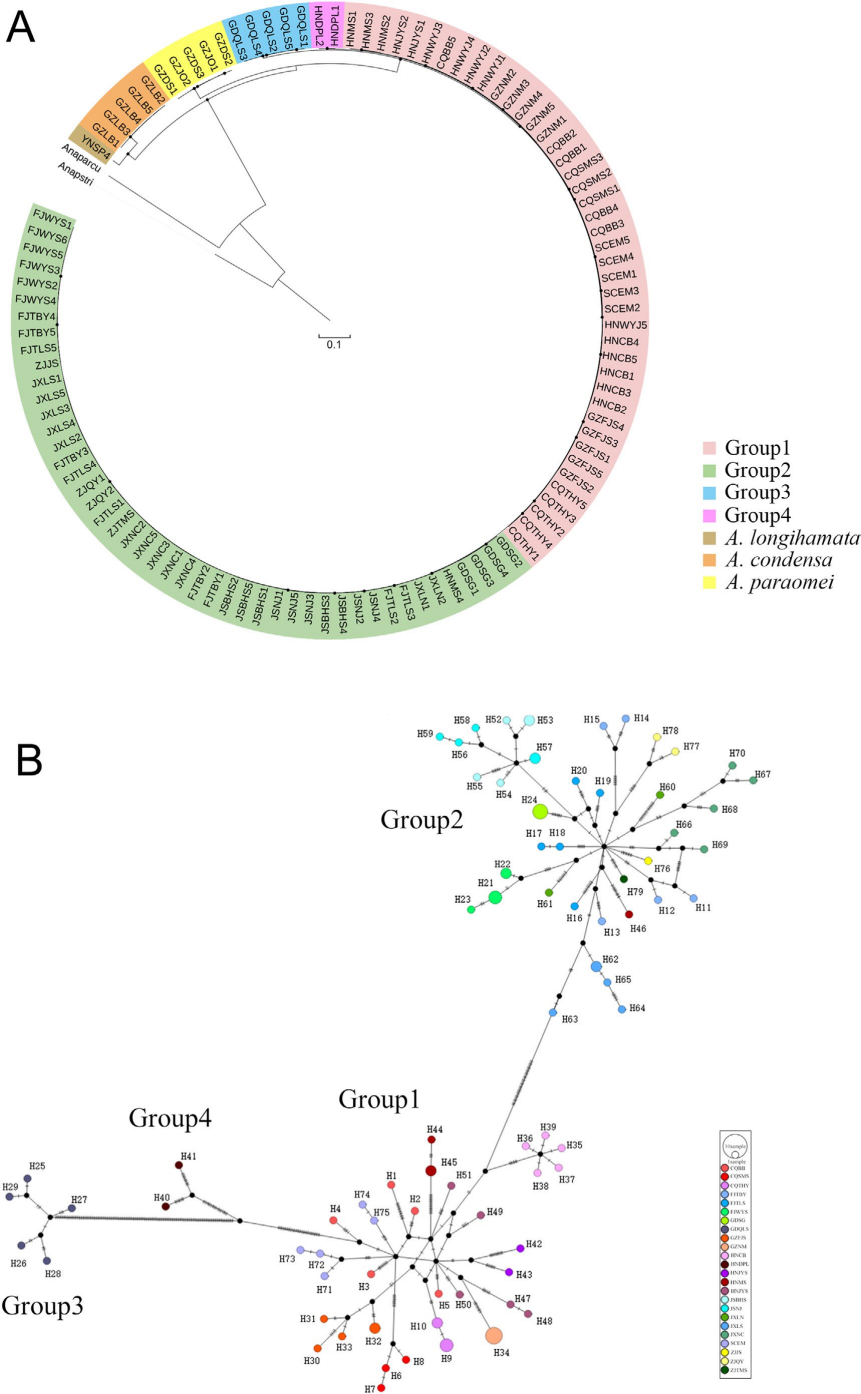
Maximum‐likelihood phylogeny and haplotype network of *Anaplecta omei* inferred from the MG dataset. (A) Maximum‐likelihood phylogeny, “Anaparcu” and “Anapstri” represent the out‐group taxa: *Anaplecta arcuata* and *Anaplecta strigata*, respectively. Black dots denote nodes with 100% bootstrap values. (B) Haplotype network. Each short bar represents a single mutational step, and small black dots indicate unsampled (missing) haplotypes. Circle size is proportional to the number of sampled individuals sharing each haplotype.

For the MG dataset, our Maximum likelihood and Bayesian phylogenetic analyses yielded almost identical, strongly supported topologies across different groups. (Figure 2A and Figures S3, S4). Four well‐defined groups were recovered, consistent with BAPS clustering (Group 1–4). Populations from the same locality clustered together to form one single branch, showing obvious phylogeographic structure.

The haplotype network based on MG dataset (Figure 2B) showed that the population structure was similar to that recovered in the phylogenetic analyses, with four groups clearly distinguished. No haplotypes were shared among geographic populations; haplotypes clustered strictly by group. Group 3 (GDQLS population) and Group 4 (HNDPL population) exhibited the largest numbers of mutational steps, whereas Group 1 and Group 2 showed fewer. The absence of shared haplotypes and the long mutational distances among groups underscore the pronounced genetic differentiation within *A. omei*.

### 3.3 Divergence Time Estimates

**FIGURE 3:**
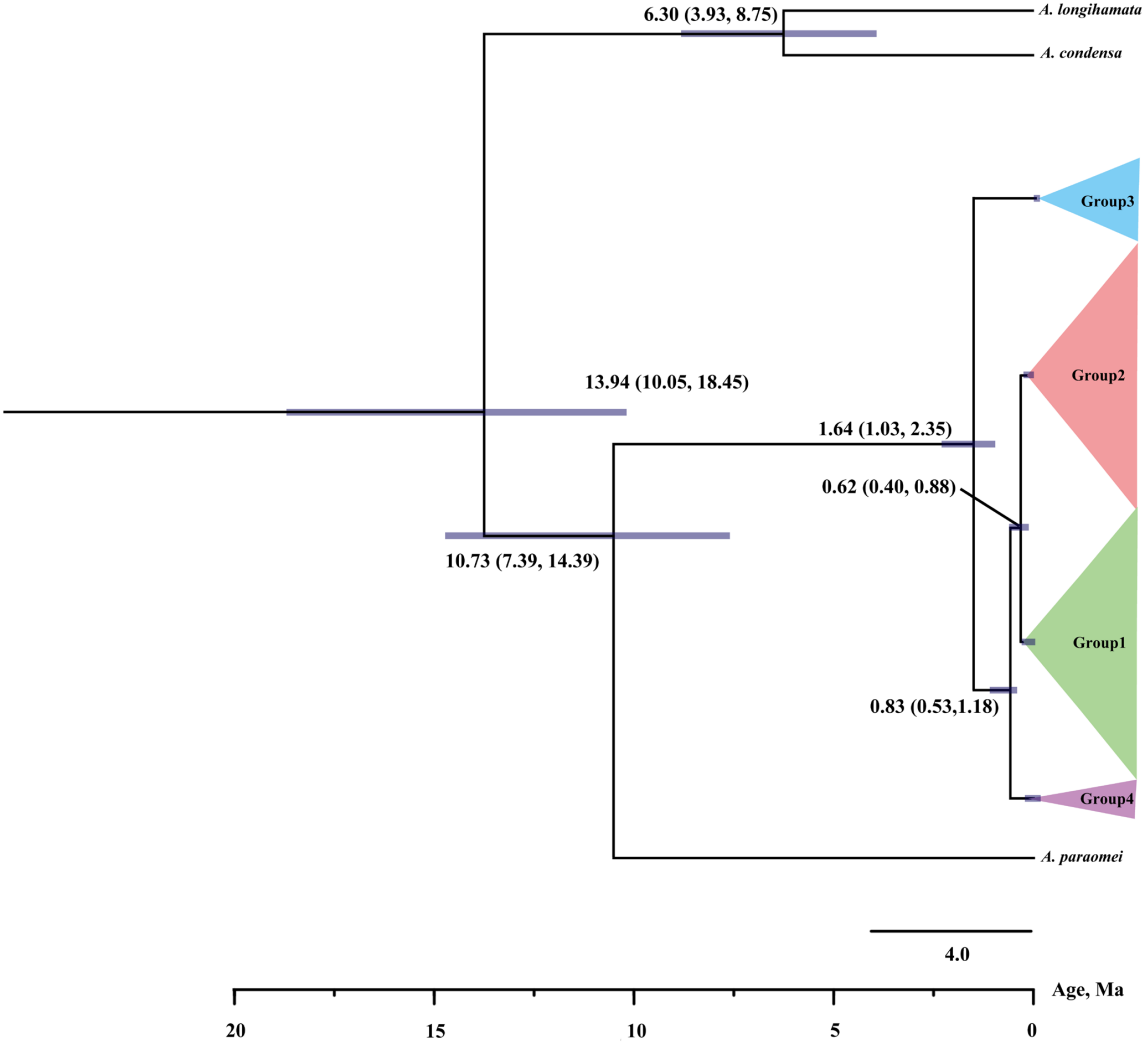
Time‐calibrated phylogeny of *Anaplecta omei* Groups inferred from the MG dataset. Purple bars denote 95% highest posterior density intervals for node ages; numbers at nodes indicate mean divergence times in millions of years ago (Ma).

The time‐calibrated phylogeny (Figure 3) dated the divergence between the clade *A. longihamata* + *A. condensa* and the clade *A. omei* + *A. paraomei* at 13.94 Ma (95% credibility interval, 10.05–18.45 Ma) in the Middle Miocene. Within the former clade, *A. longihamata* and *A. condensa* split at 6.30 Ma (95% CI, 3.93–8.75 Ma), whereas *A. omei* and its sister species *A. paraomei* diverged at 10.73 Ma (95% CI, 7.39–14.39 Ma), both during the Late Miocene.

Among *A. omei*, Group 3 separated first, at 1.64 Ma (95% CI: 1.03–2.35 Ma) in the Early Pleistocene. Group 4 diverged from the remaining two groups at 0.83 Ma (95% CI: 0.53–1.18 Ma), and the final split between Group 1 and Group 2 occurred at 0.62 Ma (95% CI: 0.40–0.88 Ma) (Figure 3).

### 3.4 Population Historic Dynamics of *A. omei*

**FIGURE 4:**
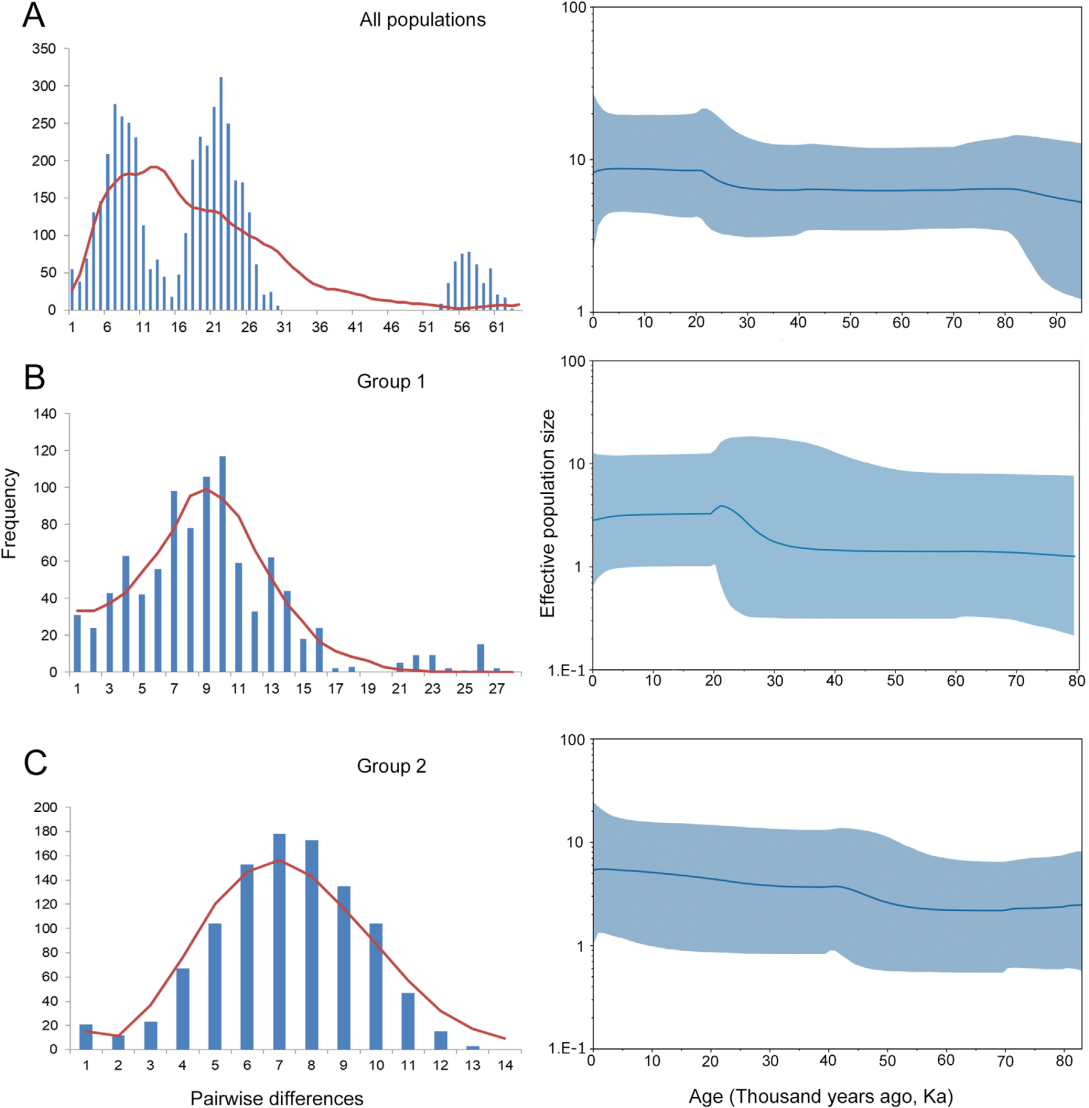
Mismatch distributions and Bayesian skyline plots for *Anaplecta omei*. Panels: (A) all populations combined, (B) Group 1, and (C) Group 2. Left‐hand sub‐panels: Blue histograms show the observed pairwise‐difference frequencies, and the red curve represents the distribution expected under a sudden‐expansion model. Right‐hand sub‐panels: The blue line traces the mean effective population size through time, and the blue shaded band indicates the 95% highest posterior density interval.

Neutrality tests (Table 6) showed significantly negative Tajima's *D* and Fu's *Fs* values for the total population of *A. omei* were all significantly negative, implying a recent demographic expansion. A low raggedness index (*r*) and the unimodal results of mismatch distribution (Figure 4) further supported this scenario. Within groups, both Group 1 and Group 2 exhibited the same signature—significantly negative Tajima's *D* and Fu's *Fs*, small *r* values, and unimodal mismatch curves—indicating recent growth. τ estimates placed the onset of expansion at ca 0.038 Ma for the Group 1 and 0.041 Ma for the Group 2 (≈38–41 ka). Bayesian skyline plots corroborated these results: Group 1 showed a pronounced increase in effective population size ~20–30 ka, followed by a slight decline after 20 ka, whereas Group 2 expanded ~40–50 ka and has continued a gradual increase thereafter. Sample sizes for Group 3 and Group 4 were insufficient for reliable neutrality or mismatch analyses.

**TABLE 6:** Neutral detection and mismatch distribution parameters of different groups of *A. omei*.

Group	Tajima's *D*	Fu's *Fs*	SSD	*r*
ALL	−1.71292**	−24.63700***	0.01292	0.00344
Group 1	−1.99382***	−10.39305***	0.00462	0.127241
Group 2	−2.12632***	−18.62602***	0.01523**	0.012498
Group 3	−1.04849	−0.18500	0.00628	0.050000
Group 4	—	—	—	—

*Note:* *significant, *p < 0.05, **p < 0.01, ***p < 0.001. (Tajima 1989; Fu 1997).

## 4 Discussion

### 4.1 Genetic Diversity and Differentiation Analysis

Across all four groups of *A. omei* and most sampling localities, high haplotype diversity (*Hd*) combined with low nucleotide diversity (*π*) suggested a recent demographic expansion from a limited ancestral gene pool. Among adequately‐sampled populations (≥ 5 individuals), HNMS (Mang Mountain) showed the highest *π* with similarly elevated *Hd*, consistent with a relatively older and more stable population fostered by the area's mild climate and rich floristic diversity. Located at the junction of southern and central subtropical floras, Mang Mountain likely provided ecological conditions that fostered long‐term accumulation of genetic variation in *A. omei*. Populations CQBB, FJTBY, and FJTLS also harbored relatively high diversity, which may be maintained by intermittent gene flow from neighboring regions (Slatkin 1987). By contrast, the apparently high *Hd* and *π* values for HNJYS, HNDPL, and JXLN are likely artifacts of small sample sizes. Finally, no genetic variation was detected in GZNM or GDSG, a pattern that could reflect extensive intra‐population gene flow, or the fact that the sampled individuals may have originated from the same ootheca, given their proximal collection sites.

### 4.2 Population Genetic Structure

BAPS clustering resolved four well‐defined groups in *A. omei*, mirroring the structure recovered by both the phylogenetic tree and the haplotype network and pointing to pronounced group differentiation. AMOVA showed 77% of the total genetic variance resides among groups, confirming marked divergence. The observed pattern is largely shaped by regional topography: Group 1 is restricted to the Sichuan Basin and Guizhou Plateau, whereas Group 2 occupies the hilly southeast and the middle–lower Yangtze Plain. These regions are separated by the Wushan and Xuefeng Mountains, which likely act as major dispersal barriers for *A. omei*, as documented for other insects (Liu et al. 2018: *Hyalessa maculaticollis*; Zhang et al. 2019: *Lycorma delicatula*; Du et al. 2020: *Dendrolimus punctatus*) and for a variety of animal taxa (Slatkin 1993: gull and pocket gopher; Ye et al. 2013: *Paa spinosa* ). Consistency between branch‐model tests—which detected no group‐specific differences in selection—and the strong geographic signal suggests that isolation by physical barriers, rather than adaptive divergence, is the primary driver of the observed structure. High pairwise *Fst* values combined with low *N*_*m*_ estimates further indicate the restricted gene flow among most populations—a restriction exacerbated by rugged terrain, habitat heterogeneity, and the species' aggregative behavior, low vagility, and limited effective dispersal. Collectively, geographic distance, complex landscape, and poor dispersal capacity interact to shape the genetic architecture of *A. omei*.

### 4.3 Phylogenetic Relationships and Divergence Time

The dated phylogeny resolved four strongly supported intraspecific groups encompassing 24 populations, revealing a clear phylogeographic structure in *A. omei*. Regional uplift of the Tibetan Plateau triggered major shifts in regional temperature, atmospheric circulation, and precipitation—especially during the Late Miocene–Pliocene (ca. 3.50 Ma), when peripheral ranges such as the Hengduan Mountains were formed (Fang 2005; Sun et al. 2011; Wang et al. 2014). Such geological upheavals commonly foster species diversification. Divergence‐time estimates indicate that the clade containing *A. paraomei* + *A. omei* split from *A. longihamata* + *A. condensa* at approximately 13.94 Ma, a period corresponding to continued plateau uplift. Subsequently, *A. longihamata* separated from *A. condensa* at ~6.30 Ma, and *A. paraomei* diverged from *A. omei* at ~10.73 Ma, coinciding with further development of the Hengduan Mountains.

Within *A. omei* itself, group differentiation began in the Early Pleistocene (~1.64 Ma), which may have coincided with the Qingzang Orogeny, and continued during or following the Wangkun Glaciation (0.50–0.70 Ma), yielding the split between Group 1 and Group 2 at ~0.62 Ma. Subsequent divergences within Group 1 occurred around 0.40 Ma, shortly after the Zhonglianggan Glaciation (0.46–0.52 Ma). The early divergence of the Mang Mountain population exemplifies this phase. Mountain barriers such as the Nanling Mountains have reinforced these splits, as illustrated by genetic discontinuity between Mang Mountain (north of the Nanling Mountains) and Shaoguan (south of the Nanling Mountains). Overall, tectonic uplift, glacial oscillations, and intricate local topography collectively shaped the diversification of *A. omei* and its congeners by impeding gene flow, promoting isolation, and facilitating genetic drift.

### 4.4 Population Historic Dynamics

Although the Quaternary ice sheets were restricted to high‐latitude regions of North America and Europe, the associated glacial stages brought colder and drier conditions to unglaciated China (Zhou et al. 2004; Liu et al. 2012). Such climatic deterioration typically forced species into lower‐latitude or micro‐refugia, restricting gene flow and preserving distinct ancestral haplotypes (Willis et al. 2004). In *A. omei*, the absence of shared or central haplotypes among groups, together with the star‐like branches within Group 1 and Group 2 in the haplotype network, indicates that these groups persisted in separate refugia and expanded independently following the retreat of glacial climates.

Multiple lines of evidence support this scenario: neutrality tests, mismatch‐distribution analyses, and Bayesian skyline plots all point to post‐glacial expansion. It should be noted that Bayesian skyline plots do not directly estimate census population size, but rather infer effective population size (*Ne*) based on mitochondrial variation. In *A. omei*, *Ne* may be several orders of magnitude lower than census size due to inheritance patterns and demographic stochasticity; nevertheless, the temporal trends revealed by this analysis remain informative. The skyline plot reveals a pronounced increase in effective population size for Group 2 at ~40–50 ka, coinciding with an intensified monsoon that delivered ample precipitation (Zhao et al. 2007) and likely facilitated dispersal. In contrast, Group 1 expanded mainly between ~20–30 ka before plateauing after ~20 ka, coincident with the onset of cooler, drier conditions during the Last Glacial Maximum (LGM) (~18 ka). Similar climate‐driven demographic slowdowns have been documented in other taxa (Li et al. 2009; Tian et al. 2010; Qu et al. 2011; Zhang et al. 2015; Xue et al. 2019) and appear to have limited demographic expansion in *A. omei*.

It should also be noted that our inferences are based solely on mitochondrial genomes and uneven sampling among groups; future studies incorporating nuclear markers and denser sampling, especially for Group 3 and Group 4, will be essential to corroborate and refine these demographic reconstructions.

## 5 Conclusions

This study presents the first comprehensive phylogeographic analysis of *A. omei*, using extensive geographic sampling and complete mitochondrial genomes. We detected high haplotype diversity but low nucleotide diversity across populations, and a clear phylogeographic structure comprising four deeply differentiated groups that diverged mainly during the Pleistocene. Strong among‐group differentiation, low gene flow, concordant clustering, and the lack of group‐specific shifts in selective pressure indicate that geographic barriers and Quaternary climate oscillations, rather than adaptive divergence, primarily shaped this structure. Our results highlight how topography and Pleistocene climate jointly act to promote divergence in a low‐dispersal cockroach. Future research incorporating denser sampling with nuclear and epigenomic data will further refine the reconstructions of *A. omei*'s evolutionary history, refugial dynamics, and dispersal corridors.

## Author Contributions

**Tunan Zhou:** conceptualization (equal), data curation (lead), formal analysis (equal), investigation (equal), methodology (equal), visualization (lead), writing – original draft (lead). **Jing Zhu:** conceptualization (equal), formal analysis (equal), investigation (equal), methodology (equal), visualization (supporting). **Chenhui Cao:** data curation (supporting), visualization (supporting). **Yanli Che:** conceptualization (equal), methodology (equal), resources (equal), writing – review and editing (equal). **Zongqing Wang:** conceptualization (lead), data curation (equal), formal analysis (equal), funding acquisition (lead), investigation (lead), methodology (equal), project administration (lead), resources (equal), supervision (equal), writing – review and editing (equal).

## Funding

This work was supported by the National Natural Science Foundation of China, 32170458. National Key Research and Development Program of China, 2022FY202100.

## Conflicts of Interest

The authors declare no conflicts of interest.

## Supporting information


**Appendix S1:** ece373086‐sup‐0001‐AppendixS1.docx.

## Data Availability

The data that support the findings of this study are openly available in GenBank under accessions numbers PX672859 to PX672966. The raw sequencing reads generated in this study have been deposited in the NCBI Sequence Read Archive (SRA) database under the BioProject accession number PRJNA1413994.
