# Supplementary material for: Pleistocene Climate Oscillations and Geographic Barriers Shaped the Phylogeographic Structure of Anaplecta omei (Blattodea, Blattoidea, Anaplectidae) in Southern China: Evidence From Mitochondrial Genomes
Source: Ecol Evol. 2026 Feb 12;16(2):e73086. doi: 10.1002/ece3.73086 (PMC12900622; doi:10.1002/ece3.73086)
Supplement: Supplementary file 1 — Appendix S1: ece373086‐sup‐0001‐AppendixS1.docx. [file ECE3-16-e73086-s001.docx]

**TABLES**

**Table S1:** Assembly statistics of the mitochondrial genomes of *Anaplecta*.

| **Population** |  | **Organism** | **Assembly**  **length (bp)** | **Coverage**  **or read**  **depth (X)** | **GC%** | **Accession Number** |
| --- | --- | --- | --- | --- | --- | --- |
| CQBB1 |  | *Anaplecta omei* | 15348 | 546 | 30.4 | PX672859 |
| CQBB2 |  | *Anaplecta omei* | 15349 | 516 | 30.4 | PX672860 |
| CQBB3 |  | *Anaplecta omei* | 15348 | 677 | 30.4 | PX672861 |
| CQBB4 |  | *Anaplecta omei* | 15348 | 1259 | 30.4 | PX672862 |
| CQBB5 |  | *Anaplecta omei* | 15350 | 537 | 30.4 | PX672863 |
| CQSMS1 |  | *Anaplecta omei* | 15348 | 390 | 30.4 | PX672864 |
| CQSMS2 |  | *Anaplecta omei* | 15348 | 482 | 30.4 | PX672865 |
| CQSMS3 |  | *Anaplecta omei* | 15348 | 473 | 30.4 | PX672866 |
| CQTHY1 |  | *Anaplecta omei* | 15347 | 315 | 30.4 | PX672867 |
| CQTHY2 |  | *Anaplecta omei* | 15347 | 235 | 30.4 | PX672868 |
| CQTHY3 |  | *Anaplecta omei* | 15347 | 644 | 30.4 | PX672869 |
| CQTHY4 |  | *Anaplecta omei* | 15347 | 236 | 30.4 | PX672870 |
| CQTHY5 |  | *Anaplecta omei* | 15347 | 469 | 30.4 | PX672871 |
| FJTBY1 |  | *Anaplecta omei* | 15351 | 193 | 30.4 | PX672872 |
| FJTBY2 |  | *Anaplecta omei* | 15351 | 1617 | 30.4 | PX672873 |
| FJTBY3 |  | *Anaplecta omei* | 15350 | 1007 | 30.4 | PX672874 |
| FJTBY4 |  | *Anaplecta omei* | 15348 | 1077 | 30.4 | PX672875 |
| FJTBY5 |  | *Anaplecta omei* | 15348 | 1208 | 30.4 | PX672876 |
| FJTLS1 |  | *Anaplecta omei* | 15349 | 292 | 30.5 | PX672877 |
| FJTLS2 |  | *Anaplecta omei* | 15350 | 274 | 30.4 | PX672878 |
| FJTLS3 |  | *Anaplecta omei* | 15350 | 447 | 30.4 | PX672879 |
| FJTLS4 |  | *Anaplecta omei* | 15349 | 357 | 30.4 | PX672880 |
| FJTLS5 |  | *Anaplecta omei* | 15349 | 296 | 30.4 | PX672881 |
| FJWYS1 |  | *Anaplecta omei* | 15348 | 1117 | 30.4 | PX672882 |
| FJWYS2 |  | *Anaplecta omei* | 15349 | 1329 | 30.4 | PX672883 |
| FJWYS3 |  | *Anaplecta omei* | 15348 | 2093 | 30.4 | PX672884 |
| FJWYS4 |  | *Anaplecta omei* | 15349 | 1415 | 30.4 | PX672885 |
| FJWYS5 |  | *Anaplecta omei* | 15348 | 1714 | 30.4 | PX672886 |
| FJWYS6 |  | *Anaplecta omei* | 15349 | 156 | 30.4 | PX672887 |
| GDSG1 |  | *Anaplecta omei* | 15349 | 636 | 30.4 | PX672888 |
| GDSG2 |  | *Anaplecta omei* | 15349 | 1076 | 30.4 | PX672889 |
| GDSG3 |  | *Anaplecta omei* | 15349 | 480 | 30.4 | PX672890 |
| GDSG4 |  | *Anaplecta omei* | 15349 | 1396 | 30.4 | PX672891 |
| GDQLS1 |  | *Anaplecta omei* | 15365 | 584 | 30.4 | PX672892 |
| GDQLS2 |  | *Anaplecta omei* | 15365 | 472 | 30.4 | PX672893 |
| GDQLS3 |  | *Anaplecta omei* | 15365 | 419 | 30.4 | PX672894 |
| GDQLS4 |  | *Anaplecta omei* | 15356 | 629 | 30.5 | PX672895 |
| GDQLS5 |  | *Anaplecta omei* | 15365 | 520 | 30.4 | PX672896 |
| GZDS1 |  | *Anaplecta paraomei* | 15356 | 659 | 30.8 | PX672897 |
| GZDS2 |  | *Anaplecta paraomei* | 15356 | 952 | 30.8 | PX672898 |
| GZDS3 |  | *Anaplecta paraomei* | 15356 | 1221 | 30.8 | PX672899 |
| GZFJS1 |  | *Anaplecta omei* | 15348 | 689 | 30.4 | PX672900 |
| GZFJS2 |  | *Anaplecta omei* | 15348 | 368 | 30.4 | PX672901 |
| GZFJS3 |  | *Anaplecta omei* | 15348 | 269 | 30.4 | PX672902 |
| GZFJS4 |  | *Anaplecta omei* | 15348 | 905 | 30.4 | PX672903 |
| GZFJS5 |  | *Anaplecta omei* | 15348 | 299 | 30.4 | PX672904 |
| GZJO1 |  | *Anaplecta condensa* | 15356 | 1431 | 30.8 | PX672905 |
| GZJO2 |  | *Anaplecta condensa* | 15356 | 434 | 30.8 | PX672906 |
| GZLB1 |  | *Anaplecta condensa* | 15354 | 792 | 31.1 | PX672907 |
| GZLB2 |  | *Anaplecta condensa* | 15355 | 725 | 31.1 | PX672908 |
| GZLB3 |  | *Anaplecta condensa* | 15355 | 556 | 31.1 | PX672909 |
| GZLB4 |  | *Anaplecta condensa* | 15357 | 988 | 31.1 | PX672910 |
| GZLB5 |  | *Anaplecta condensa* | 15355 | 667 | 31.1 | PX672911 |
| GZNM1 |  | *Anaplecta omei* | 15349 | 441 | 30.4 | PX672912 |
| GZNM2 |  | *Anaplecta omei* | 15349 | 294 | 30.4 | PX672913 |
| GZNM3 |  | *Anaplecta omei* | 15349 | 343 | 30.4 | PX672914 |
| GZNM4 |  | *Anaplecta omei* | 15349 | 256 | 30.4 | PX672915 |
| GZNM5 |  | *Anaplecta omei* | 15349 | 338 | 30.4 | PX672916 |
| HNCB1 |  | *Anaplecta omei* | 15348 | 280 | 30.4 | PX672917 |
| HNCB2 |  | *Anaplecta omei* | 15348 | 293 | 30.4 | PX672918 |
| HNCB3 |  | *Anaplecta omei* | 15351 | 479 | 30.4 | PX672919 |
| HNCB4 |  | *Anaplecta omei* | 15349 | 478 | 30.4 | PX672920 |
| HNCB5 |  | *Anaplecta omei* | 15350 | 464 | 30.4 | PX672921 |
| HNDPL1 |  | *Anaplecta omei* | 15347 | 684 | 30.4 | PX672922 |
| HNDPL2 |  | *Anaplecta omei* | 15348 | 620 | 30.4 | PX672923 |
| HNJYS1 |  | *Anaplecta omei* | 15350 | 387 | 30.4 | PX672924 |
| HNJYS2 |  | *Anaplecta omei* | 15350 | 937 | 30.4 | PX672925 |
| HNMS1 |  | *Anaplecta omei* | 15348 | 182 | 30.4 | PX672926 |
| HNMS2 |  | *Anaplecta omei* | 15348 | 329 | 30.4 | PX672927 |
| HNMS3 |  | *Anaplecta omei* | 15348 | 241 | 30.4 | PX672928 |
| HNMS4 |  | *Anaplecta omei* | 15350 | 211 | 30.4 | PX672929 |
| HNWYJ1 |  | *Anaplecta omei* | 15350 | 661 | 30.4 | PX672930 |
| HNWYJ2 |  | *Anaplecta omei* | 15351 | 690 | 30.4 | PX672931 |
| HNWYJ3 |  | *Anaplecta omei* | 15348 | 600 | 30.4 | PX672932 |
| HNWYJ4 |  | *Anaplecta omei* | 15348 | 519 | 30.4 | PX672933 |
| HNWYJ5 |  | *Anaplecta omei* | 15348 | 497 | 30.4 | PX672934 |
| JSBHS1 |  | *Anaplecta omei* | 15351 | 229 | 30.4 | PX672935 |
| JSBHS2 |  | *Anaplecta omei* | 15351 | 681 | 30.4 | PX672936 |
| JSBHS3 |  | *Anaplecta omei* | 15348 | 657 | 30.4 | PX672937 |
| JSBHS4 |  | *Anaplecta omei* | 15351 | 394 | 30.4 | PX672938 |
| JSBHS5 |  | *Anaplecta omei* | 15350 | 622 | 30.4 | PX672939 |
| JSNJ1 |  | *Anaplecta omei* | 15349 | 182 | 30.4 | PX672940 |
| JSNJ2 |  | *Anaplecta omei* | 15351 | 283 | 30.4 | PX672941 |
| JSNJ3 |  | *Anaplecta omei* | 15349 | 407 | 30.4 | PX672942 |
| JSNJ4 |  | *Anaplecta omei* | 15349 | 475 | 30.4 | PX672943 |
| JSNJ5 |  | *Anaplecta omei* | 15349 | 341 | 30.4 | PX672944 |
| JXLN1 |  | *Anaplecta omei* | 15349 | 1199 | 30.3 | PX672945 |
| JXLN2 |  | *Anaplecta omei* | 15350 | 467 | 30.4 | PX672946 |
| JXLS1 |  | *Anaplecta omei* | 15349 | 769 | 30.4 | PX672947 |
| JXLS2 |  | *Anaplecta omei* | 15349 | 439 | 30.4 | PX672948 |
| JXLS3 |  | *Anaplecta omei* | 15351 | 715 | 30.4 | PX672949 |
| JXLS4 |  | *Anaplecta omei* | 15349 | 662 | 30.4 | PX672950 |
| JXLS5 |  | *Anaplecta omei* | 15349 | 826 | 30.4 | PX672951 |
| JXNC1 |  | *Anaplecta omei* | 15351 | 500 | 30.4 | PX672952 |
| JXNC2 |  | *Anaplecta omei* | 15349 | 788 | 30.4 | PX672953 |
| JXNC3 |  | *Anaplecta omei* | 15350 | 649 | 30.4 | PX672954 |
| JXNC4 |  | *Anaplecta omei* | 15349 | 865 | 30.4 | PX672955 |
| JXNC5 |  | *Anaplecta omei* | 15349 | 875 | 30.4 | PX672956 |
| SCEM1 |  | *Anaplecta omei* | 15348 | 266 | 30.3 | PX672957 |
| SCEM2 |  | *Anaplecta omei* | 15348 | 876 | 30.4 | PX672958 |
| SCEM3 |  | *Anaplecta omei* | 15348 | 219 | 30.3 | PX672959 |
| SCEM4 |  | *Anaplecta omei* | 15367 | 270 | 30.4 | PX672960 |
| SCEM5 |  | *Anaplecta omei* | 15366 | 286 | 30.4 | PX672961 |
| YNSP4 |  | *Anaplecta longihamata* | 15353 | 1253 | 31.4 | PX672962 |
| ZJJS |  | *Anaplecta omei* | 15351 | 490 | 30.4 | PX672963 |
| ZJQY1 |  | *Anaplecta omei* | 15351 | 399 | 30.4 | PX672964 |
| ZJQY2 |  | *Anaplecta omei* | 15350 | 398 | 30.4 | PX672965 |
| ZJTMS |  | *Anaplecta omei* | 15352 | 1313 | 30.4 | PX672966 |

Note: Average read number: 23835314. Average read length: 149 bp. The average nucleotide composition of *A. omei* was 41.0% A, 19.1% C, 11.3% G, and 28.6% T. The average nucleotide composition of *A. paraomei* was 41.1% A, 19.6% C, 11.3% G, and 28.0% T. The average nucleotide composition of *A. condensa* was 41.0% A, 19.1% C, 11.3% G, and 28.6% T. The nucleotide composition of *A. longihamata* was 40.9% A, 19.9% C, 11.5% G, and 27.7% T.

**Table S2:** Partitions and models used in selection pressure analyses

| Dataset | Optimal Data Partitioning | Optimal Model | Sequence Length (bp) |
| --- | --- | --- | --- |
| PCG | *ATP6_c3, Cytb_c3* | GTR | 602 |
|  | *ND2_c12, ATP8_c3, COII_c12, CytB_c12, ATP6_c12* | HKY+I | 2392 |
|  | *ATP8_c12* | HKY | 104 |
|  | *ND6_c3, ND3_c3, COII_c3, COIII_c3* | TRN+G | 774 |
|  | *COI_c12, COIII_c12* | K81UF+I | 1546 |
|  | *ND2_c3, COI_c3* | HKY+I+G | 852 |
|  | *ND4_c3, ND1_c3, ND4L_c3, ND5_c3* | HKY+I+G | 1426 |
|  | *ND1_c12, ND5_c12* | HKY+I | 1772 |
|  | *ND6_c12, ND3_c12* | TIM+I | 570 |
|  | *ND4L_c12, ND4_c12* | HKY | 1080 |

**Table S3:** Partitions and models used in Bayesian Skyline Plot analyses of *A. omei*

| Lineages | Optimal Data Partitioning | Optimal Model | Sequence Length (bp) |
| --- | --- | --- | --- |
| ALL | *12S, ND5_c12* | K81UF+I | 1893 |
|  | *16S* | K81UF+I | 1266 |
|  | *22tRNA* | HKY+I+G | 1432 |
|  | *COII_c12, CytB_c12, ND2_c12, ATP6_c12* | TVM+I+G | 2340 |
|  | *ATP8_c12* | HKY | 104 |
|  | *COI_c12, COIII_c12* | K81UF+I | 1546 |
|  | *ND1_c12* | HKY+I | 626 |
|  | *ND6_c12, ND3_c12* | TIM+I | 570 |
|  | *ND4L_c12, ND4_c12* | HKY | 1080 |
| Group 1 | *12S, 16S* | K81UF+I | 2013 |
|  | *22tRNA* | TRN | 1432 |
|  | *ND6_c12, ND3_c12, ND2_c12, ATP6_c12* | TRN | 1700 |
|  | *ATP8_c12* | F81 | 104 |
|  | *COI_c12, COIII_c12* | TRN+I | 1546 |
|  | *COII_c12, CytB_c12* | HKY | 1210 |
|  | *ND1_c12, ND4_c12, ND4L_c12* | HKY+I | 1706 |
|  | *ND5_c12* | TRN+I+G | 1146 |
| Group 2 | *12S, 16S* | HKY | 2013 |
|  | *22tRNA* | HKY | 1432 |
|  | *COII_c12, ND3_c12, ATP6_c12, CytB_c12, ND2_c12* | HKY+I+G | 2574 |
|  | *ATP8_c12, ND6_c12* | HKY | 440 |
|  | *COI_c12, COIII_c12* | K81UF+I | 1546 |
|  | *ND1_c12, ND5_c12* | HKY+I | 1772 |
|  | *ND4L_c12, ND4_c12* | HKY | 1080 |

**Table S4:** AMOVA analysis results of *A. omei* based on BAPS grouping.

| Source of variation | Degrees of freedom, (d.f.) | Sum of squares | Variance components | Percentage  of variation | Fixation Indices |
| --- | --- | --- | --- | --- | --- |
| Among groups | 3 | 1787.417 | 31.2223 | 77.02 | *Fct*=0.7702 |
| Among populations | 20 | 498.563 | 5.1400 | 12.68 | *Fsc*=0.5519 |
| Within populations | 73 | 304.700 | 4.1740 | 10.30 | *Fst*=0.8970 |

**FIGURES**


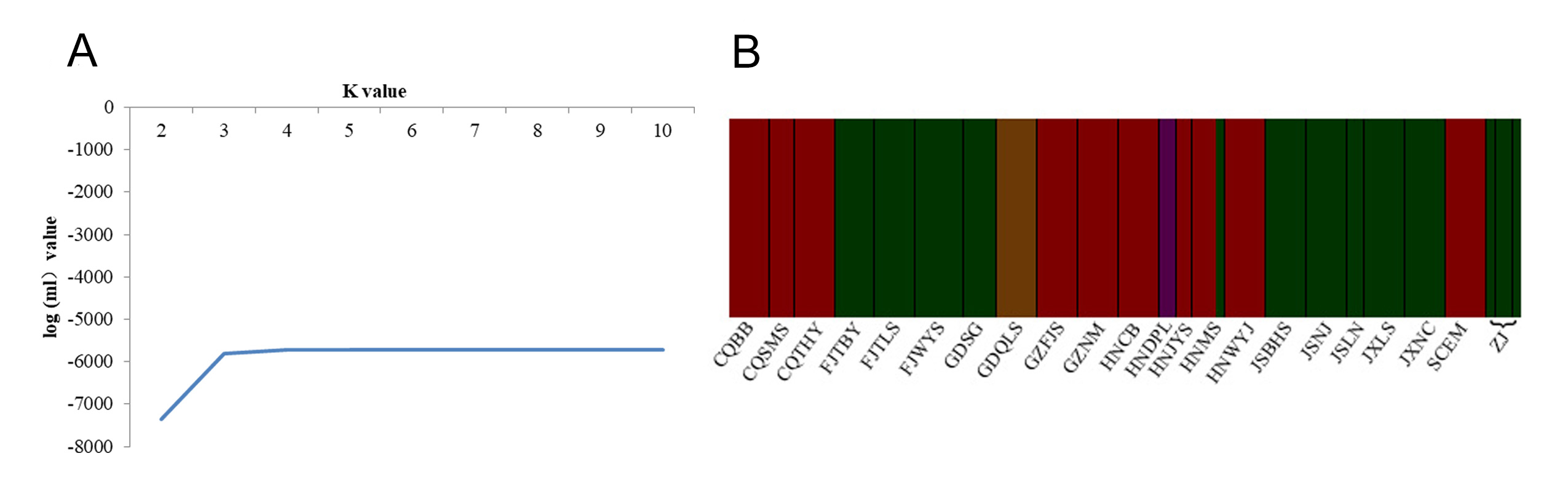


**Figure S1:** Results of BAPS analysis for 24 geographical populations of *A. omei*

(A) Variation in the optimal log marginal likelihood (log(ml)) values across different preset population groupings (*K* values) in the BAPS analysis. The log(ml) values stabilized at *K*=4, indicating the optimal grouping. (B) Results of BAPS clustering: Populations sharing the same color belong to the same group. The ZJ cluster includes three geographical populations: ZJJS, ZJQY, and ZJTMS.


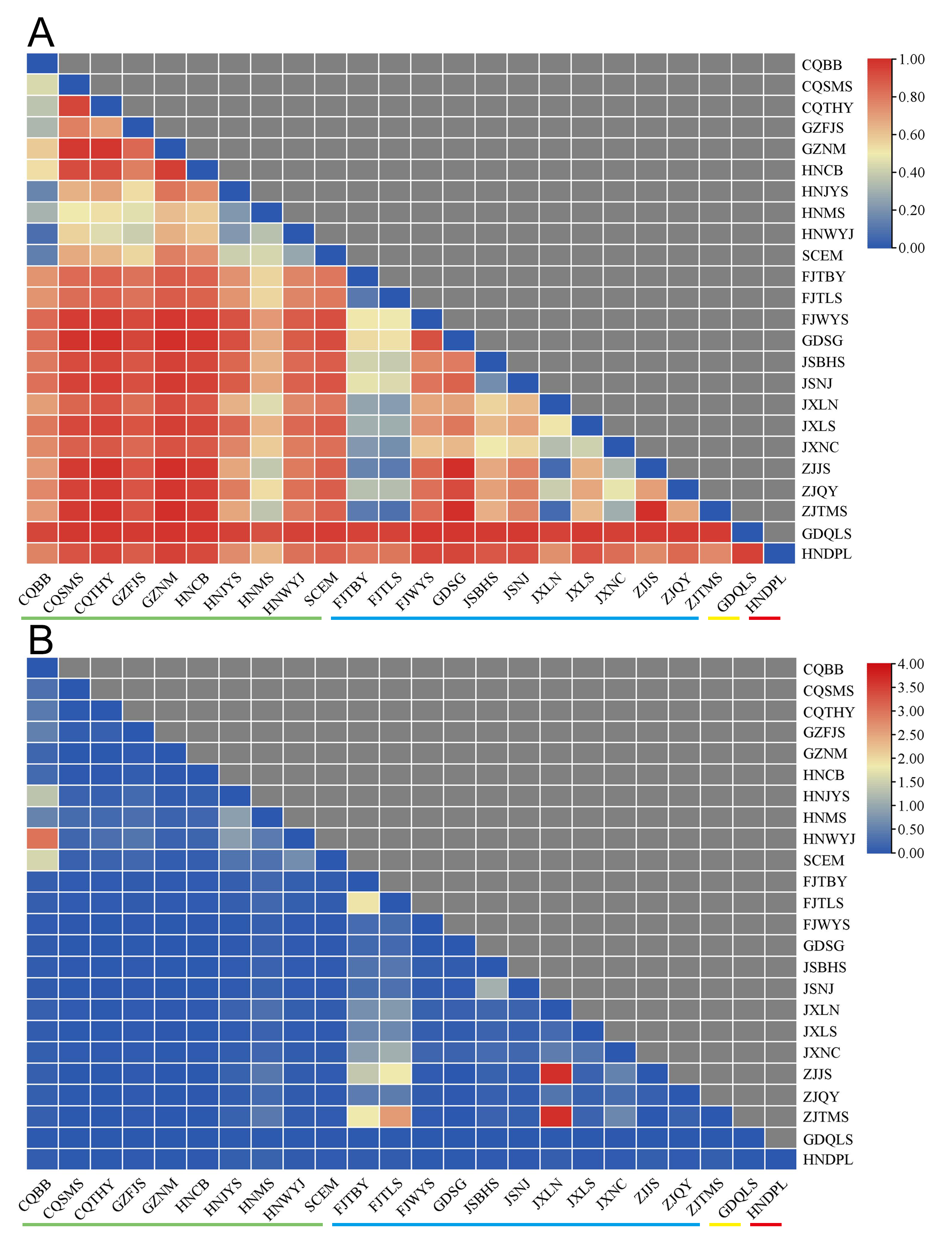


**Figure S2:** Pairwise genetic differentiation index (*Fst*) and gene flow *(N_m_*) among geographical populations of *A. omei*

(A) Heatmap of genetic differentiation index (*Fst*); (B) Heatmap of gene flow (*N_m_*). The green, blue, yellow, and red rectangles represent geographical populations belonging to Group 1, Group 2, Group 3, and Group 4, respectively. The geographical populations on the x-axis and y-axis are arranged in the same order in both panels.


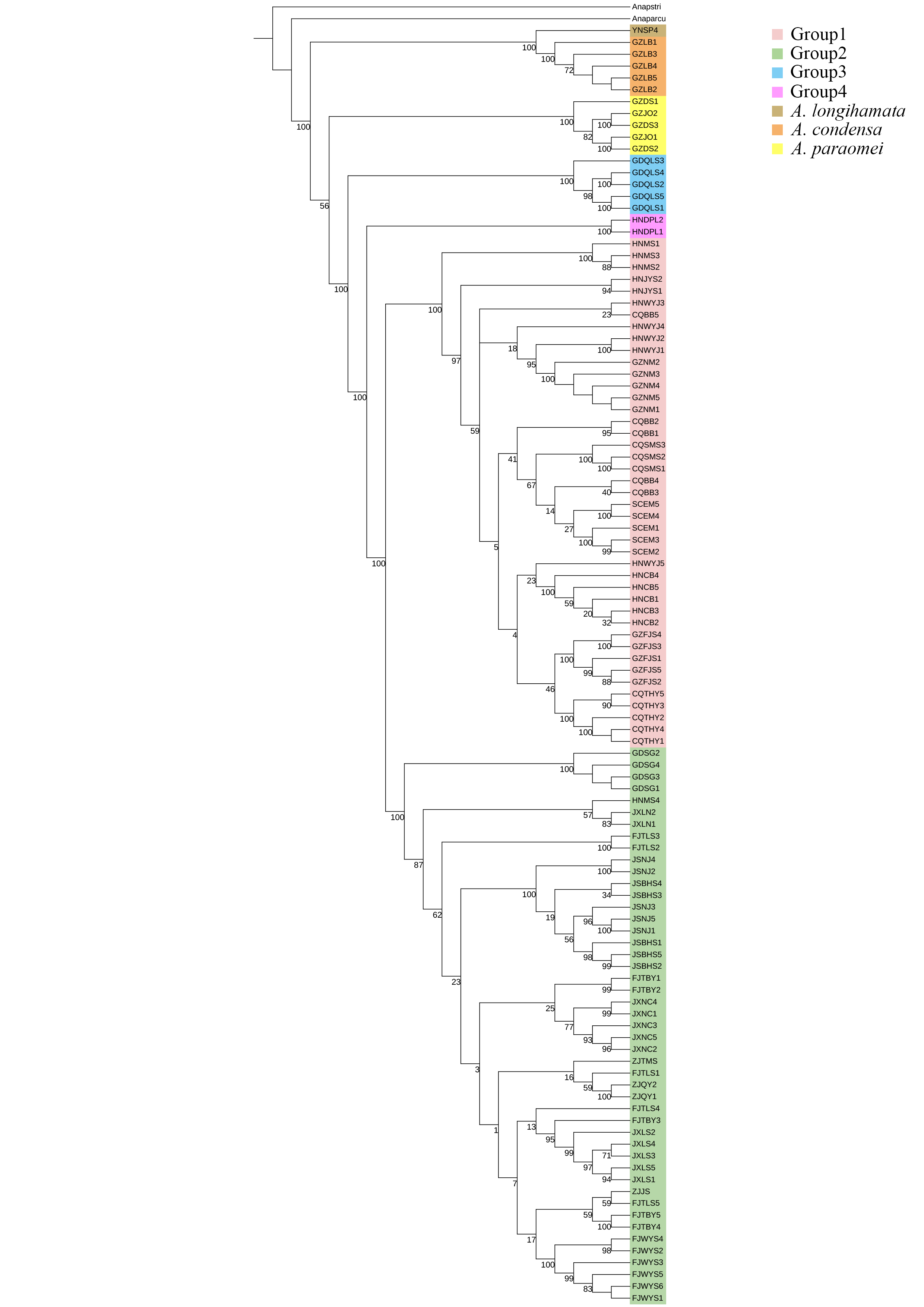


**Figure S3:** Maximum-likelihood phylogeny of *A. omei* was with bootstrap support values reconstructed based on the MG dataset, “Anaparcu” and “Anapstri” represent the out-group taxa: *Anaplecta arcuata* and *Anaplecta strigata*, respectively.


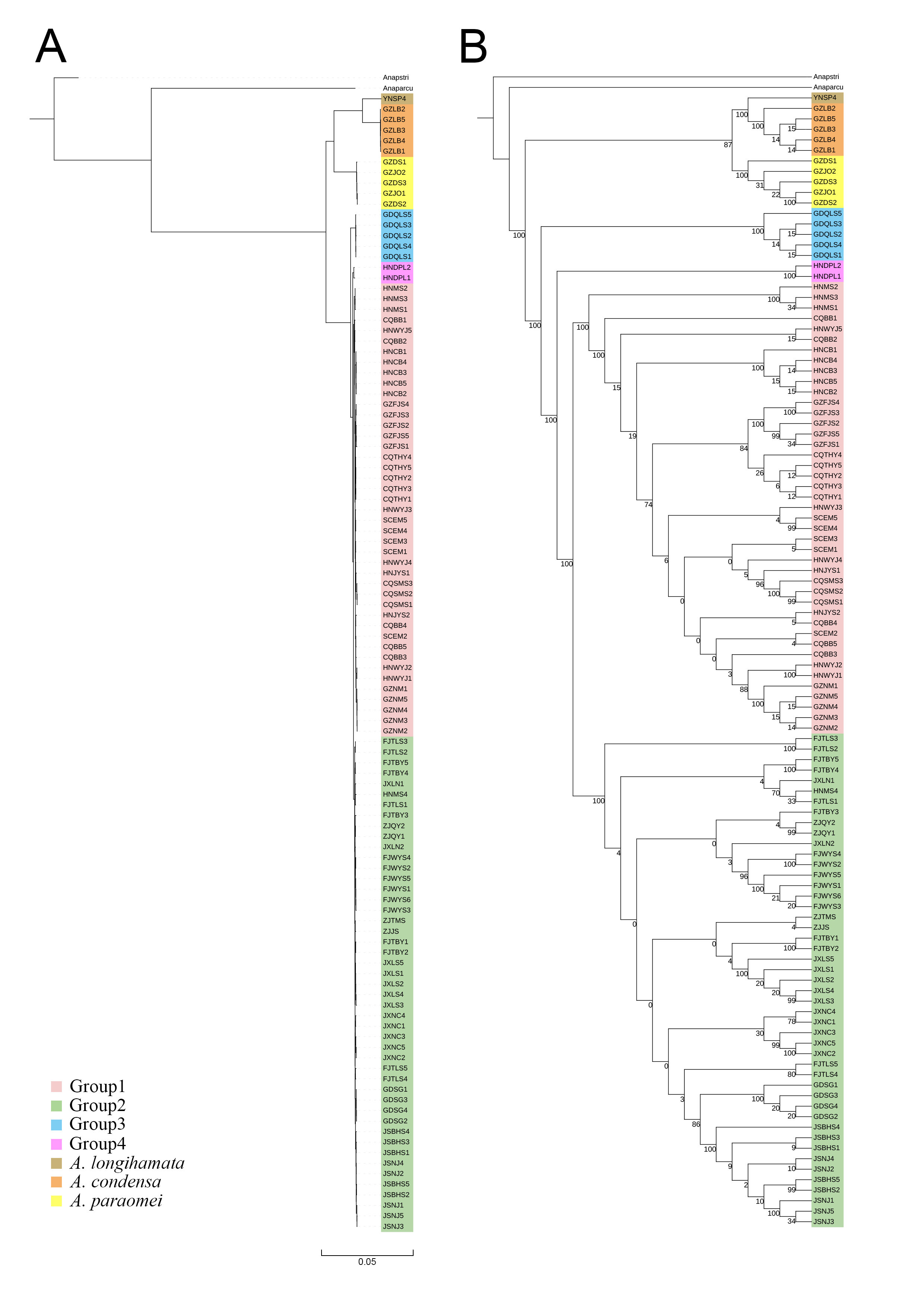


**Figure S4:** Bayesian inference phylogeny of *A. omei* was reconstructed based on the MG dataset, “Anaparcu” and “Anapstri” represent the out-group taxa: *Anaplecta arcuata* and *Anaplecta strigata*, respectively. (A) BI tree showing branch lengths. (B) BI tree showing posterior probability support values.
